# Quantitative analyses of adiposity dynamics in zebrafish

**DOI:** 10.1080/21623945.2019.1648175

**Published:** 2019-08-14

**Authors:** Loes M.H. Elemans, Iris Pruñonosa Cervera, Susanna E. Riley, Rebecca Wafer, Rosalyn Fong, Panna Tandon, James E.N. Minchin

**Affiliations:** Centre for Cardiovascular Science, University of Edinburgh, Edinburgh EH16 4TJ, UK

**Keywords:** zebrafish, adipose tissue, animal models

## Abstract

Adipose tissues often exhibit subtle, quantitative differences between individuals, leading to a graded series of adiposity phenotypes at the population level. Robust, quantitative analyses are vital for studying these differences. In this Commentary we highlight two articles from our lab that employ sensitive new methods in zebrafish capable of delineating complex and quantitative adiposity phenotypes. In the first article, we utilized in vivo imaging to systematically quantify zebrafish adipose tissues. We identified 34 regionally distinct zebrafish adipose tissues and developed statistical models to predict the size and variance of each adipose tissue over the course of zebrafish growth. We then employed these models to identify effects of strain and diet on adipose tissue growth. In the second article, we employed deep phenotyping to study complex disease-related adiposity traits. Using this methodology, we identified that adipose tissues have unique capacities to re-deposit lipid following food restriction and re-feeding. These distinct re-deposition potentials led to widespread fat distribution changes following re-feeding. We discuss how these novel findings may provide relevance to health conditions such as anorexia nervosa. Together, the strategies described in these two articles can be used as unbiased and quantitative methods to uncover new relationships between genotype, diet and adiposity.

## Commentary

### Adiposity is a quantitative trait and a key determinant of disease risk

The worldwide prevalence of obesity is increasing dramatically, and has resulted in a concomitant increase in obesity-associated diseases such as type 2 diabetes and cardiovascular disease [1,2]. Obesity is characterised by excessive accumulation of lipid within adipose tissues (AT) and subsequent AT dysfunction [3,4]. ATs are specialized lipid-accumulating structures that primarily come in two distinct types: white and brown [5]. White adipose tissues (WAT) have a range of diverse functions; however, a primary role is to regulate systemic energy homeostasis by releasing lipid into, or sequestering lipid from the circulation [5]. Brown adipose tissue (BAT) is thermogenic and utilises stored lipid to generate heat [6]. Obesity is highly heritable suggesting a large genetic component [7], and, at a population level, adiposity levels (composed primarily of WAT) are highly variable and form a continually graded series from one adiposity extreme to the other [8]. Genome-wide association studies (GWAS) have begun to identify the polygenic genetic architecture underlying adiposity [9–11]. However, as adiposity is a complex, quantitative trait, individual underlying mechanisms may only account for relatively small, subtle effects [12,13]. Traditional model systems to study adiposity often suffer from low statistical power and an inability to detect small differences [14]. Therefore, new model systems are needed that facilitate quantitative analysis of adiposity with large sample sizes.

### The regional distribution of adipose tissues is a strong predictor of disease

In addition to absolute AT levels, the regional distribution of ATs is also a strong predictor of disease susceptibility. ATs are distributed throughout the body [5,15,16]. In humans, ATs are primarily found in subcutaneous locations or within the abdominal cavity [5,17], although increasing functional importance is being established for bone marrow AT [18]. The regional location of AT influences disease risk. For example, accumulation and dysfunction of WAT around the abdomen, primarily surrounding visceral organs (VAT), is strongly associated with metabolic disease risk [19,20]. Conversely, lower-body gluteofemoral subcutaneous WAT (SAT) and abdominal SAT have been shown to protect against metabolic dysfunction [20–30]. Therefore, the regional distribution of WAT influences disease risk, and accordingly, proportionality between WATs (e.g., the ratio of upper to lower body WAT or the ratio of VAT to SAT) are strong predictors for the future development of metabolic disease [31,32]. It would be highly advantageous to develop a model system with the ability to quantify all ATs in an individual and ascertain in an unbiased manner how inter-AT relationships change and influence disease susceptibility [20]. This need is typified by proportionality changes between distinct WATs having unique and distinct influences on disease [33,34]. Owing to these inter-AT relationships, it is important to assess adipose attributes (e.g., size, shape, location etc) relative to other ATs. In more traditional model systems used to study adiposity, it is typically difficult to image whole-animal adiposity at reasonable resolution and with large enough sample sizes to detect small effects. Therefore, new model systems are needed to study inter-AT relationships and interactions.

### Zebrafish is an innovative model system to assess quantitative traits such as adiposity levels and adipose distribution

Zebrafish provide a tractable, evolutionarily conserved and optically transparent model system for whole-animal and quantitative measurement of adiposity. Zebrafish AT is morphologically homologous to mammal WAT [35–37]. Zebrafish AT expresses specific markers of mammalian WAT, including *pparg, cebpa* and *fabp11a* (the zebrafish ortholog of mammalian *FABP4*) [35,37–39]. Further, zebrafish AT appears to function in an equivalent manner to mammalian WAT; mobilizing lipid in response to food restriction and accumulating increased lipid in response to caloric overload [37,40,41]. As zebrafish do not regulate their body temperature, current dogma suggests that zebrafish do not possess thermogenic BAT [16]; however, this has not been fully assessed. Intriguingly, a conserved *uncoupling protein 1* (*ucp1*) gene is present in ectothermic teleosts (including zebrafish) [42,43], and warm acclimated liver mitochondria from carp exhibit a GDP-sensitive and fatty acid-inducible proton leak reminiscent of Ucp1-mediated uncoupling in mammalian mitochondria [44]. It is completely unknown whether zebrafish possess beige adipocytes, which reflects the nascent state of AT research in zebrafish. Mammalian WAT secretes an array of hormones (adipokines) including Adiponectin, Leptin, Resistin, TNFa, IL6, and aP2 [45]. The presence and role of adipokines in zebrafish AT is largely unstudied. *adiponectin* is expressed by zebrafish adipocytes; however, a functional role in zebrafish is unknown [35]. Zebrafish possess two *leptin* genes [46], and a function for Leptin as an ‘adipostat’ does not appear to be conserved in zebrafish [47,48]. Thus, although some aspects of WAT biology are understudied in zebrafish, it is clear that WAT is highly conserved in zebrafish and fulfils similar functions to mammalian WAT. In addition to biomedical impact, modelling WAT biology in zebrafish may provide insights relevant to the aquaculture industry [49]. To summarize, modelling adiposity traits in zebrafish is likely to provide information relevant concerning mammalian WAT with the potential to lead to new insights for therapeutic intervention.

### *In vivo* imaging with fluorescent lipophillic dyes can be used to quantify adipose tissues in zebrafish

**Figure 1.**
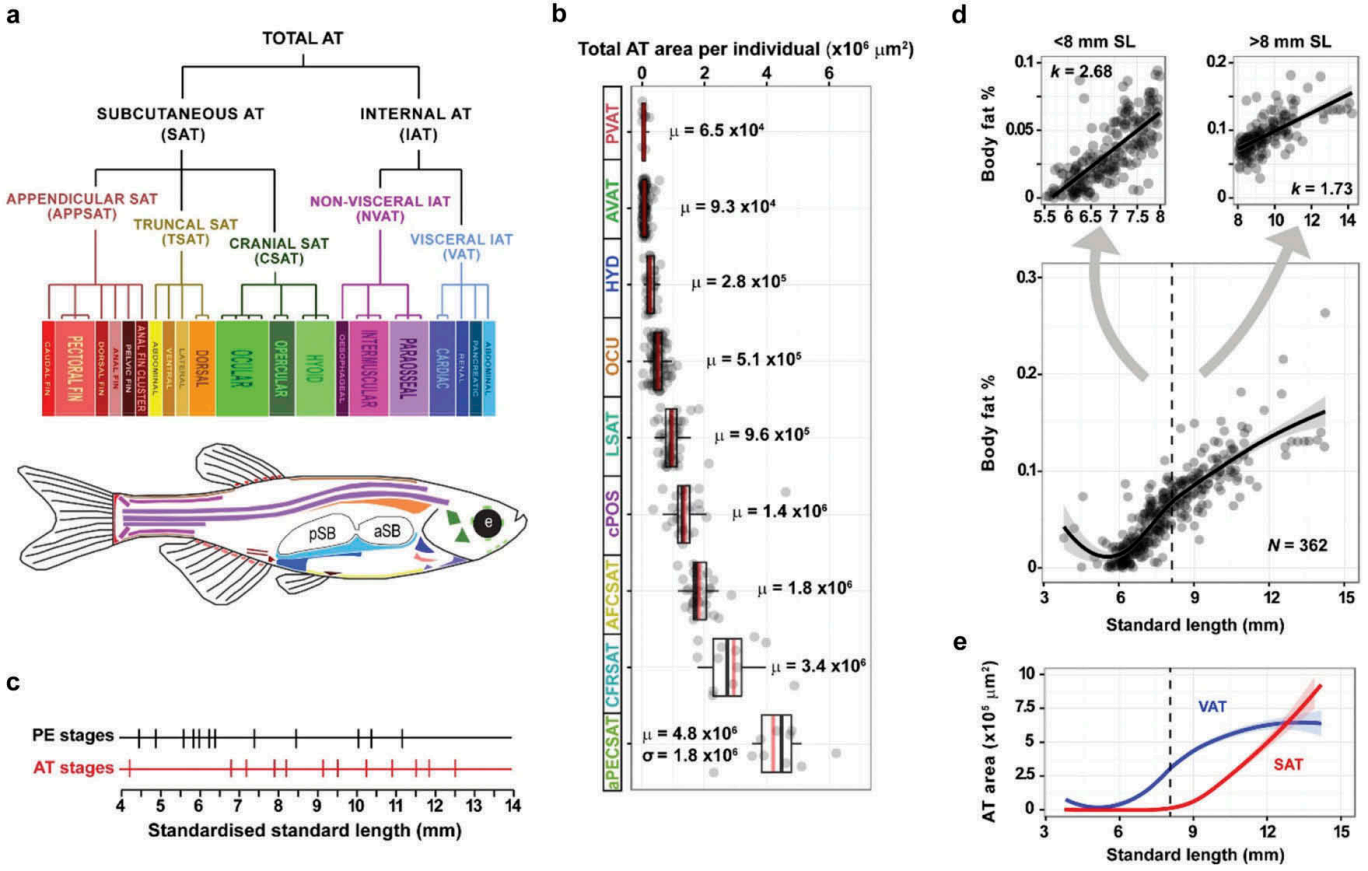
**Quantitative analysis of adiposity in zebrafish. a**. Tree documenting the 34 regionally distinct adipose tissues and their anatomical relatedness. See Minchin et al., (2017)[51] for further details. Total adipose tissue is first divided into internal (IAT) and subcutaneous (SAT) adipose. Schematic of zebrafish details anatomic location of each adipose tissue (colour coded). b. Quantification of the increase in total adipose area as zebrafish grow. The nine categories (PVAT, AVAT, HYD, OCU, LSAT, cPOS, AFCSAT, CFRSAT and aPECSAT) represent post-embryonic stages of zebrafish development as detailed in Minchin et al., (2017) [51]. c. Representation of the standardised standard lengths at which established post-embryonic (PE) stages have been identified, together with the adipose tissue (AT) stages identified in Minchin et al., (2017) [51]. d. Graph showing body fat % relative to standard length. Note the change in the slope of the line from ~8 mm standard length (lower graph). Upper graphs show datapoints from <8 mm (upper left graph) or >8 mm (upper right graph). Note the decrease in allometric growth rate (k) in fish >8 mm. These data show that the rate of body fat accumulation slows after 8 mm SL coincident with the appearance and diversification of SAT. e. Graph showing the temporal dynamics of VAT and SAT accumulation in zebrafish. Note, prior to 8 mm SL VAT exhibits substantial growth, which plateaus at ~8 mm SL coincident with the appearance of SAT.

ATs are highly dynamic organs, which are unique to bony vertebrates [16]. In mammals WAT is first formed during embryonic development [16]. In both mice and humans, SAT development is first detected in the embryo, while VAT develops postnatally [50]. In zebrafish, the development of AT differs with VAT first forming in the post-embryonic fish at around 14 dpf (days post fertilisation), before SAT appears later [40,51] (Figure 1). Once AT has been deposited in zebrafish, it expands to form a vast array of adipose depots at anatomically distinct locations [40,51] (Figure 1a). Quantification of total AT reveals how extensively adipose expands in zebrafish (Figure 1b). To quantify AT distribution, we recently published an article that characterised 34 adipose depots present in zebrafish and described AT growth characteristics [40,51]. This study provides a standard classification system and nomenclature of zebrafish AT to support the use of this animal model in AT research (Figure 1a). Specifically, the classification system was adapted from a previous system used in human AT classification [17]. In our study, fluorescent lipophilic dyes (FLDs) and whole-animal *in vivo* imaging were used to identify, quantify and classify AT depots in zebrafish, according to their anatomical location [40,51]. Extensive information on use of FLDs can be found in Minchin and Rawls [40,51]. Use of FLDs and imaging on a stereoscope allows rapid imaging and large sample sizes. However, for more detailed (higher resolution) analyses like confocal imager, smaller sample sizes are used. We verified that FLD-labelled AT is an accurate proxy for triacylglyceride (TAG) content [41,52] and, as proof-of-principle, FLD+ areas of three distinct ATs (PVAT, AVAT and CFRSAT) were measured and then dissected and quantified for TAG content by a fluorimetric assay [51]. We found that FLD+ area was an accurate predictor for TAG mass for each AT (R^2^ = 0.78, 0.87 and 0.82; all P < 0.0001), which supported the method of area measurements of individual ATs as an accurate estimator of lipid content. These methods are relatively fast to undertake and large sample sizes were achievable [51,53]. Therefore, FLDs allow the large-scale collection of quantitative adiposity data.

### Generation of a classification system to study zebrafish adipose tissues

To facilitate the study of inter-AT relationships, we used FLD-based imaging to generate a comprehensive anatomical classification system for zebrafish ATs. We found that total AT in zebrafish was divided into internal AT (IAT) and subcutaneous ATs (SAT) (Figure 1a) [40,51]. IAT can be associated with internal visceral organs (visceral IAT, VAT) or not (non-visceral IAT, NVAT) and can be divided in pancreatic VAT (PVAT), abdominal VAT (AVAT), renal VAT (RVAT) and cardiac VAT (CVAT) depending on the associated organ (Figure 1a). PVAT is the first adipose tissue to form in the zebrafish, being deposited at around 4.2 mm in fish length (called standard length; SL). After the initiation of PVAT development, it undergoes uninhibited growth until RVAT is first detected. After the formation of RVAT, at ~8 mm SL, IAT expansion slowed (Figure 1d & e). In contrast to this growth profile, SAT did not appear until ~6 mm SL and was then observed to undergo uninhibited expansion throughout the remainder of the data set (Figure 1d & e) [51]. SAT is located between the dermis and the aponeuroses and fasciae of the muscles and can be associated with the trunk (truncal SAT, TSAT), head (cranial SAT, CSAT) and fins (appendicular SAT, APPSAT). SAT can be divided into lateral SAT (LSAT) running laterally along the trunk, dorsal sat (DSAT) in the dorsal-most trunk and ventral SAT (VSAT) between the anal and caudal fins. CSAT Is the first SAT to appear in zebrafish and can be found in the operculum (OPC), eye (ocular CSAT, OCU) and hyoid apparatus (HYD). APPSAT can be divided in pelvic fin SAT (PELSAT), anal fin ray SAT (AFRSAT), dorsal fin ray (DFRSAT), anal fin cluster SAT (AFCSAT), caudal fin ray SAT (CFRSAT) and pectoral fin SAT (PECSAT) (Figure 1a). The identification of these ATs, together with predictions for when they appear, allowed us to define new AT-related post-embryonic staging criteria (Figure 1c). Taken together, this classification system will facilitate analysis of inter-AT relationships and their response to dietary and genetic manipulation.

### Quantitative models help predict the expected sizes of zebrafish adipose tissues

In addition to a classification system, we also generated statistical models to predict AT size and variance. Quantification of the size of each AT along with the developmental stage at which they appear allowed us to apply logistical regression models that accurately predict the time of AT appearance and size throughout development [51]. These statistical models will facilitate analysis of potentially small deviations following genetic or dietary manipulations. We found that new ATs appeared throughout development and gave a steady increase in total AT area (Figure 1b). However, growth within each AT occurred at different rates and ATs appeared at different developmental milestones (Figure 1b) [51]. Apart from in urohyoid AT (UHD), SL could predict over 50% of the size variance in all depots (mean R^2^ = 0.84), suggesting that the size of ATs is a function of SL [51]. Age was the best predictor of UHD size (R^2^ = 0.44) with other measures of zebrafish size failing to reach this level of prediction [51]. SL had a reduced predictive ability (mean R^2^ = 0.51) in other ventral ATs (e.g. hyoid apparatus adipose), suggesting possible experimental error in their measurement [51]. Tracking of body fat % reveals that prior to reaching 8 mm SL, zebrafish undergo a rapid accumulation of body fat primarily in VAT (Figure 1d). This is supported by tracking the times of AT development which showed that VAT appears first and rapidly expands before slowing as SAT appears and diversifies (Figure 1e). Therefore, a model can be proposed whereby zebrafish initially deposit lipid in VAT during rapid increases in body fat (Figure 1d & E). However, from ~8 mm SL, zebrafish slow the rate of body fat accumulation and begin storing lipid in diverse SAT sites (Figure 1d & e). These interesting dynamics suggest that VAT may be beneficial for post-embryonic development and survival, and further suggest that ~8 mm SL is a critical size for the zebrafish to reach before accumulation of body fat % slows. Altogether, the predictive power of these statistical models will facilitate robust analyses of quantitative adiposity traits in zebrafish.

### Applying quantitative *in vivo* imaging to analyse diet and strain effects on adiposity in zebrafish

Following the generation of a classification system and statistical models to study zebrafish adiposity, as a proof-of-principle, we also examined the effects of diet and genotype. A high-fat diet (HFD) supplement was introduced to increase the adiposity of our specimens and to evaluate the utility of the AT classification system [51]. The supplement, 5% chicken egg yolk (CEY), was provided to 27 dpf zebrafish in addition to the standard feed during 14 days in daily incubations of 2 hours. After the supplementation, total AT area was increased in the HFD-exposed specimens compared to their counterparts without an increase in SL [51]. Furthermore, by applying our classification system and predictive models we identified that VAT enlarged to a greater degree following HFD than SAT, suggesting that ATs in different regions can have different responses to HFD in zebrafish [51]. The abovementioned analyses were performed in the Ekkwill (EKW) zebrafish strain. To determine whether these dynamics were comparable across genetic backgrounds, ATs from the WIK zebrafish strain were also quantified [51]. There were no differences in AT types, configuration, and timing of AT appearance between the two strains [51]. However, as seen in previous work by McMenamin et al [54], WIKs had a smaller SL at a comparable EKW developmental stage [51]. In agreement with this, WIKs at the PR developmental stage (~7.4–9.1mm SL) had smaller AT sizes [51]. However, by the pCVAT developmental stage, WIKs had reduced IAT and increased SAT relative to EKWs [51]. Intriguingly, although fat distribution was altered between the strains, no difference was observed in total AT [51]. Taken together, these experiments demonstrate the utility of the classification system and predictive models to discern complex and subtle AT distribution differences in zebrafish and in response to diet manipulation and genetic differences.

### Deep phenotyping and phenomics to study complex, quantitative traits

Deep phenotyping, or phenomics, offers an effective strategy to comprehensively analyse quantitative adiposity traits. The phenome describes a comprehensive set of biochemical or physical traits which belong to a given organism [55]. Deep phenotyping refers to the measurement of phenomes as they change in response to genetic mutation and environmental influences [56]. Deep phenotyping has been successfully used in zebrafish to identify skeletal deformations [57,58]. However, deep phenotyping has so far not been applied to complex adiposity phenotypes. Current phenotyping of organisms is typically limited to the focus of the individual scientist, while advocates of phenomics argue that phenotypic data should instead be collected at multiple levels of granularity, from behavioural phenotypes to cell specific changes [59,60]. Phenomic level data is thought to be necessary to understand how genetic variants affect phenotypes and to provide raw data to decipher the causes of complex disease [55]. Furthermore, phenomics allow the determination of links between genotypes, environmental factors and phenotypes resulting in the formation of the genome-phenome map, which is related to the multiple ways genotypic information influences the phenotype of an organism [55]. Another aim of phenomics is to understand the genetic regulation of complex traits [61]. GWAS have identified many genetic associations with disease, but these associations do not account for all of the observed phenotypic variance [62]. One example of this is human height, where nearly 200 validation loci explain only 10% of the genetic variation. The ‘missing heritability’ can be associated with thousands of single nucleotide polymorphisms (SNPs), which are either very rare or have very small effects at the population level [63]. It has been suggested that traditional disease risk factors such as weight and blood chemistry are more powerful predictors of disease than SNP associations [64,65]. Therefore, increased levels of phenotypic data provided by deep phenotyping may be useful in disease prediction, particularly with subtle and quantitative adiposity traits.

### Applying deep phenotyping to study quantitative adiposity traits in zebrafish

There have been multiple efforts to map the phenome of various model organisms, and a zebrafish phenome project was proposed in 2010 [60]. We recently published an article which applied deep phenotyping techniques to assess adiposity changes in zebrafish [53]. Our goal was to use the quantitative methods of zebrafish AT identification described previously [40,51] and couple this to deep phenotyping to analyse complex responses of adipose tissues to genetic and environmental perturbations [53]. After quantification of all ATs in the zebrafish, we reasoned that collecting large amounts of quantitative adiposity data could be used to classify individuals based on their adiposity profiles and could then be used to identify subtle, quantitative adiposity phenotypes. Existing data was utilized to generate post-embryonic stage-specific phenotypic profiles that capture adiposity information (adiposity profiles) [53]. From 456 zebrafish, 67 traits were quantified in each fish, including AT area measurements, measures of body size (SL and body area), composite AT groupings (total AT, VAT and SAT) and AT proportionality assessments (VAT:SAT ratios). Correlations were computed between each trait and assessed to determine how adiposity relationships and patterns change in fish of distinct stages [53]. As expected, considerable differences in adiposity profiles at distinct stages were observed. For example, PVAT was initially positively correlated with SL (stage PB), before becoming progressively more inversely correlated with SL in larger fish (stage SA) [53]. Therefore, we reason that adiposity profiles capture dynamic changes in adiposity patterns at distinct developmental stages and can thus be useful indicators of ‘normal’ adiposity levels and variation.

### Multivariate statistics to identify small, quantitative adipose phenotypes in zebrafish

Linear discriminant analysis (LDA) is a data dimensionality reduction technique used for pattern classification and machine learning applications. LDA is used to maximize the variance within data and separate the data into classes based on the variance. LDA was used to classify individual fish according to expected adiposity traits. LDA assigned 87.5% of the wild-type fish into correct stages based on adiposity profiles [53]. Across multiple independent clutches, LDA resulted in an average classification rate of 84.8 ± 2.4% (mean ± SE). As proof-of-concept, zebrafish growth hormone (*gh*) mutants were used to determine if LDA can be used to detect adiposity phenotypes, as these mutants show increased adiposity relative to size-matched wild-type siblings [66]. LDA correctly identified 60% of cases based on adiposity profiles in the *gh* mutant clutch, and 36% in mixed wild-type and mutant clutches. In conclusion, we found LDA could be used to identify phenotypic adiposity traits based on adiposity profiles. To further apply LDA methodology, a small-scale ‘shelf’ screen of mutants was used to help identify novel zebrafish adipose mutants [53]. Of the eight mutant lines, *nrp2b* robustly presented mutant identification rates higher than expected from wild-type zebrafish, suggesting that *nrp2b* mutation may influence adiposity in zebrafish. Intriguingly, in a subsequent F0 screen for genes implicated in hypothalamic obesity, we also identified *nrp2b* as an important regulator of adiposity [67]. In the latter study we identified widespread increases in adiposity in *nrp2b* mutants [67]. However, the sa18942 allele exhibited specific decreases in PVAT [53]. It will be important to reconcile these apparent phenotypic differences by assessing wider aspects of adiposity in the sa18942 mutants, together with analysis of PVAT in F0 mutants. Further, the strategy for mutating *nrp2b* differed in these two studies – the sa18942 mutation is a single base-pair change in an essential splice site, whereas in the latter study we utilised multiplex CRISPR to generate larger deletions towards the translational start [53,67].

### Deep phenotyping to identify the effect of diet on zebrafish adipose tissues

**Figure 2.**
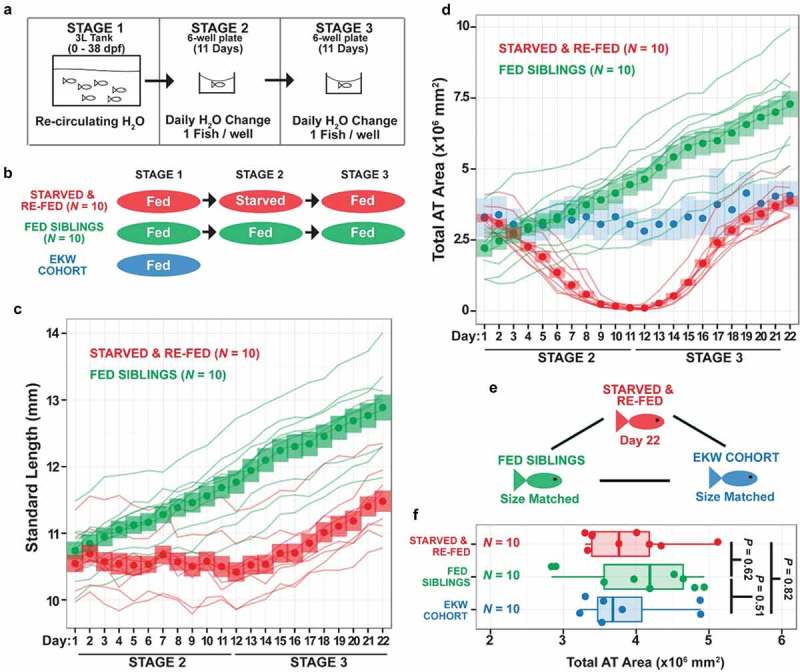
**Food restriction and re-feeding in zebrafish**. Data taken and re-plotted from Minchin et al. [53]. A. Experimental design showing food restriction (starvation) and re-feeding over the course of 22 days. B. Schematic showing the breakdown of experimental groups at each stage of the experiment. EKE cohort represents size-matched wild-type fish from a previously described cohort (Minchin & Rawls, 2017). C. Growth trajectories of continuously fed (green) and starved and re-fed siblings (red). Lines represent the growth trajectories of individual fish. Circles represent the mean standard length and coloured bars represent the standard deviation of standard lengths. As can be seen, the starved and re-fed animals slow their growth during food restriction (stage 2). D. Analysis of total AT in the starved and re-fed (red), continuously fed (green) and size-matched EKW control zebrafish (blue). As before, lines represent the growth trajectories of individual fish. Circles represent the mean standard length and coloured bars represent the standard deviation of standard lengths. Note the loss of total AT in starved and re-fed animals during stage 2, followed by re-deposition during stage 3 (re-feeding). Note also, that starved and re-fed animals ‘catch-up’ to size-matched wild-type control fish (blue). E,F. Analysis showing the equivalency in size in animals used for comparative analysis.

In Minchin et al. [53],,LDA was applied to identify diet-induced changes in adiposity (Figure 2). Zebrafish were subjected to prolonged food restriction followed by re-feeding until total adiposity levels were completely replenished (Figure 2). During the course of diet manipulation, the Nile Red stained zebrafish showed that AT-localised lipid was fully mobilised during food restriction as shown by the lack of Nile Red+ lipid within adipose tissue (Figure 2). Principal component analysis (PCA) was used to identify whether adiposity trait dynamics were different during food restriction and re-feeding, and strikingly, PCA suggested that food restriction and re-feeding elicits changes in AT distribution even after complete recovery of total AT levels. Furthermore, IATs had a more limited capacity to replenish lipid when compared to SAT in size-matched ‘continuously-fed’ animals. The average IAT recovery was ~80%, whereas SAT recovery was ~160%, leading to a reduced IAT:SAT ratio compared to size-matched ‘continuously fed’ control fish. Taken together, food restriction and re-feeding leads to altered fat distribution even after full replenishment of AT levels, which is caused by a general failure of internal ATs to replenish lipid levels leading to differences in body fat distribution.

### Anorexia nervosa – medical relevance of changes in body fat distribution following food restriction and recovery

Our findings that food restriction and re-feeding can alter body fat distribution is of potential medical importance – is there evidence for fat distribution differences following dietary intervention in humans? Anorexia nervosa is a health problem associated with reduced food intake and low body fat [68]. Recovery of body fat levels in anorexic patients is a key treatment strategy [68]. Owing to such an important medical condition, an extensive literature is present on body fat distribution both before and after weight restoration in anorexia nervosa. Intriguingly, studies of adolescent or adult females with anorexia nervosa identified different effects on fat distribution – adolescents had greater reductions in visceral adipose, whereas adults had greater reductions in subcutaneous and adipose at extremities [69–75]. Strikingly, weight restoration in adults with anorexia nervosa led to a 212% increase in subcutaneous fat and only a 117% increase in visceral fat [75]. These data show that body fat distribution changes in anorexia nervosa and following weight recovery is related to development and suggests that zebrafish may be a useful model system for assessing fat distribution changes in anorexia nervosa.

### Conclusions

To conclude, deep phenotyping offers considerable potential for the identification of parameters that signify health and disease. Particularly, deep phenotyping is important for adiposity traits which often exhibit complex and continuous phenotypes which are often inadequately assessed by qualitative methods. Quantitative methodology is essential to evaluate complex morphological and functional phenotypic changes, particularly for understanding and interpreting health and disease states. In zebrafish, we identified a striking phenotype whereby IATs do not fully replenish lipid following re-feeding, compared to total AT levels of normally fed individuals. Furthermore, SATs have a greater capacity to re-deposit lipid compared to VATs and, after re-feeding, zebrafish display decreased IAT:SAT ratios. Accumulation of SAT is associated with reduced risk of metabolic disease in humans [30] and zebrafish [76,77], raising the possibility that the increased SAT accumulation induced by long-term food restriction and refeeding may have favourable health effects. Deep phenotyping and phenomics can be used as a new method to assess adiposity profiles in zebrafish, which will be useful to identify new genetic and environmental factors governing AT development and physiology.
